# Animals, veterinarians and the sociology of diagnosis

**DOI:** 10.1111/1467-9566.13017

**Published:** 2019-10-28

**Authors:** Pru Hobson‐West, Annemarie Jutel

**Affiliations:** ^1^ School of Sociology and Social Policy University of Nottingham Nottingham UK; ^2^ Faculty of Health Victoria University of Wellington Wellington New Zealand

**Keywords:** diagnosis, veterinary, animals, professionals, ethics, species

## Abstract

While sociologists of medicine have focused their efforts on understanding human health, illness, and medicine, veterinary medical practice has not yet caught their attention in any sustained way. In this critical review article, we use insights from the sociology of diagnosis literature to explore veterinary practice, and aim to demonstrate the importance of animals to sociological understandings of health, illness and disease. As in human medicine, our analysis shows the importance of diagnosis in creating and maintaining the power and authority of the veterinary professional. However, we then explore how diagnosis operates as a kind of dance, where professional authority can be challenged, particularly in light of the complex ethical responsibilities and clinical interactions that result from the triad of professional/owner/animal patient. Finally, we consider diagnosis via the precept of entanglement, and raise the intriguing possibility of interspecies health relations, whereby decision‐making in human health care may be influenced by experiences in animal health care and *vice‐versa*. In our conclusion, we argue that this analysis provides opportunities to scholars researching diagnosis in human health care, particularly around the impact of commercial drivers; has implications for veterinary and public health practitioners; and should help animate the emerging sociology of veterinary medicine.

## Introduction: the emerging sociology of veterinary medicine

By comparison with human health, the social science of animal health and medicine is in its infancy (Brown and Nading 2019, Hobson‐West 2007, Hobson‐West and Timmons 2016, Rock *et al*. 2007). However, this situation is likely to change, given the ‘animal turn’ in social science, and attempts to de‐centre the human via explorations of the way in which species are entangled (Brown and Nading 2019), bonded in significant otherness (Haraway 2003) or can be studied via multispecies ethnography (Kirksey and Helmreich 2010). Indeed, medical anthropologists have recently argued that ‘living well with other animals is … likely among the most pressing and urgent issues of our time’ (Brown and Nading 2019: 5). These authors also identify the One Health agenda, with its focus on the interplay between human and animal health, as a key governmental response to our entangled, multispecies environments.

While cognisant of these developments and policy agendas, our own conceptual approach remains primarily ‘human‐centred’ (Rock and Babinec 2008: 341) but focuses on veterinary practice as a key site of human–animal relations. In doing so, we aim to highlight themes which have resonance across understandings of both human and animal health and illness. One of the salient features of the veterinary profession is the wide range of species and cultural contexts within which they operate. As we now illustrate, this includes, but is not limited to, small animal medicine (so called ‘companion’ animals or pets, although see Haraway 2003 on this terminology), large animal medicine (equine, zoo or ‘livestock’) and other non‐therapeutic roles (including animal welfare in the laboratory and slaughterhouse).

Small animal medicine, perhaps in advance of other areas, has encouraged sociological study using techniques and concepts well known to those who study human health and illness. For example, scholars have used conversation analysis and other video analytic techniques to study communication in the small animal consultation. Drawing on concepts developed through study of human–medical interaction, existing research focuses on how consults are structured (Everitt *et al*. 2013), the delivery of good and bad news (Stivers 1998), the phrasing of recommendations (MacMartin *et al*. 2018) and how veterinarians speak to, and for, the animals under their care (Burrow 2017, MacMartin *et al*. 2014, Roberts 2004).

Beyond communication, fewer studies have focused on identity in veterinary practice. For example, Morris (2012a) found that veterinary staff refer to animals both as subjects (patients) and objects (the client's property), whereas Burrow (2017) has shown how consultation talk works to construct animals as individuals. Anthropologist Rock and colleagues have argued that, like pet owners, veterinarians attribute ‘a personality, self or soul to their non‐human patients’ (Rock and Babinec 2008: 343). Using a historical approach, Degeling claims that this rendering of animal selfhood was influenced by the development and use of radiographic images in the clinic (Degeling 2009). Other studies have focused on the gallows humour, emotional work (Morris 2012a, 2012b) or emotional dirty work (Sanders 2010) involved in those caring for companion animals, particularly in end of life care and in death.

There is a similar concentration on questions of communication between professional and client in relation to farm animals, despite their different status from domestic animals, as means of production. Knights and Clarke (2018: 147) identify ‘shell shock’ for newly qualified vets when they first enter practice and discover that farmers are sceptical of their lack of experience. Bard *et al*. utilise role play methods to make suggestions for ways to improve interaction between vets and farmers (Bard *et al*. 2017), whereas Hamilton is concerned that the ‘illusory promises of the evidence based mantra’ is hampering effective vet–farmer communication (Hamilton 2018: 225). Other ethnographic research by the same author focuses on occupational aspects of veterinary work, and the importance of mundane objects (Hamilton 2013) and even muck (Hamilton 2007) in maintaining power relations between staff in farm veterinary clinic. As with small animal practice, research on farm animals has also focused on death. For example, Hurn and Badman‐King (2019: 141) identify a dominant veterinary ideology that killing is the ultimate form of care, and contrast this with a religious community's failed attempt to avoid euthanasia of a bull diagnosed with bovine tuberculosis.

A smaller number of sociologists have also started to explore the professional role of veterinary expertise in ‘non‐therapeutic’ contexts outside of clinical practice. For example, all animal research laboratories in the UK have a ‘Named Veterinary Surgeon’; this role demands a complicated professional identity, given a dual regulatory responsibility to promote animal welfare and help facilitate laboratory science (Ashall and Hobson‐West 2018, Hobson‐West and Davies 2018). Vets also play a key role at the point of food animal slaughter (McCabe and Hamilton 2015), in the whole governance of the food chain (Enticott *et al*. 2011) and, less well known, in facilitating the donation of companion animal blood (Ashall and Hobson‐West 2017).

Another stream of work focuses across domains of practice to explore veterinary medicine as a profession or occupation. For example, scholars have explored questions of self‐regulation and occupational closure (Hobson‐West and Timmons 2016, May 2013) the neoliberal challenge to the profession (Enticott *et al*. 2011), and even the impact of Brexit discussions on global labour flows (Enticott 2019). Others have identified a masculine gendering of veterinary practice (Clarke and Knights 2019), despite the dramatic feminisation of the profession (Allen 2016, Irvine and Vermilya 2010). Scholars in organisational studies have also highlighted professional identity struggles and significant anxiety, partly caused by the contrast between training in a positivist, objectivist science and the lived reality of work (Clarke and Knights 2018), and the marketisation common to both academia and veterinary practice (Knights and Clarke 2018).

This rich body of work is interdisciplinary in nature and is clearly aligned with topics of long‐standing and central interest to sociologists of medicine and of health, namely communication in the clinic, professional identity and prestige, regulation and the relationship with the state. Here, we seek to extend this body of work using the sociology of diagnosis, also a central concern for sociologists of health and illness. We demonstrate that the study of diagnosis shines a light on veterinary medicine in ways that reveal similarities and differences between species health/illness concerns that has the potential to be mutually informative.

In the pages which follow, we present a critical review and synthesis from several sociological sub‐fields, and also draw on contemporary documents from government websites and professional associations. Our analysis primarily focuses on the UK context, but draws on international literature, especially from North America and Australasia. Our aim is to suggest how theoretical arguments from existing sociology of diagnosis work can be productively extended to the veterinary world. As Brown and Nading have argued in relation to medical anthropology and animals, moving beyond the traditional boundaries of a discipline has the potential for ‘reorienting’ it (Brown and Nading 2019: 5). Similarly, we hope that our analysis provokes an extension of sociological arguments that are fundamental to the understanding of health, illness and medicine in animals as well as in humans.

## Diagnosis in human medical practice

Diagnosis represents a useful heuristic for understanding the potential of species ‘boundary shifting’ in the sociology of health and illness. It has played an important role in the establishment of scientific medicine. Indeed, it was via the classification of disorders that medicine made a claim for epistemic authority, so that diagnosis was not only the means to ‘penetrate the superficial appearances of a patient's troubles to the deeper roots of disease’ (Crenner 2005: 19), it was also a marker of status ‘… the climax of medical skill’, (Hadra 1902: 60) contributing to medical power. As Hadra continued ‘no one stands in higher esteem than he who is known to be a fine diagnostician’.

Its importance in the practice of medicine and the experience of illness have not eluded sociologists, who see the means by which diagnostic categories are established, the processes leading to the establishment of a diagnosis and the consequences of diagnosis labelling as fertile ground for developing new understandings of health, illness and disease (Blaxter 1978, Brown 1990, Jutel 2009). Treating diagnosis as the topic of study itself underlines both the complexity of power and of illness. From a sociological perspective, the materiality of physical illness cannot be divorced from the ways in which it is socially framed, spoken about and allocated; for diagnosis is a social good, linked to resources, status and role (Aronowitz 2008, Freidson 1972, Jutel 2011).

Diagnosis is also didactic and a tool for communication. One word or phrase summarises an extensive description: the nature of the condition, possibly its causative factors, symptoms and likely outcome. The diagnosis serves numerous social roles as it determines treatment, assigns specialism and links to prognosis.

Following Blaxter's assertions about diagnosis‐as‐category and diagnosis‐as‐process, the number of sociological studies of human diagnosis is expansive, exploring the taken‐for‐grantedness of diagnostic categories (Campos *et al*. 2006); querying the expansion of diagnostic classifications and the process by which they are standardised (Conrad 2007, Horwitz 2011, Whooley 2010); elucidating the contests present in the pursuit of diagnosis, not only between lay and professional but also between different professional categories (Gardner *et al*. 2011); underlining the mechanisms and power of the diagnostic moment (Jutel 2017, Price and Walker 2014) and so on. This body of work has also considered the impact of what Nettleton (2004) has referred to as ‘e‐scaped medicine’, whereby ‘spaces, sites and locations of the production of medical knowledge are now more diffuse and are invariably mediated by means of digital technologies’ (2004: 673). For example, interesting questions are raised by the wide availability of medical and ‘medicalesque’ information, the proliferation of self‐diagnosis tools and apps; the interference of commercial players in the diagnostic scene with profit interests; and finally, the increasingly contested nature of the diagnostic encounter.

In summary, sociologists of medicine and of health have gained much insight from a specific focus on diagnosis. We seek to apply these insights to the veterinary medical profession, and, by doing so, to set out a research agenda for future work.

## Diagnosis in veterinary medical practice

If we are to consider veterinary diagnosis in the same way that we have human diagnosis, we can see the presence of similar – and at the same time, divergent – themes. As in human medicine, we can see how veterinary professional power is partly constituted through diagnosis. As we shall see however, this takes place within an intricate set of relationships made more challenging by the contractual nature of veterinary services and by the dependent status of the animal in relation to its owner. Also, in line with human medicine, there are points of lay/expert power contention in relation to diagnosis; however, these are differently created and contested in veterinary medicine. And finally, veterinary medicine is characterised by interspecies relations, and may confront and create entanglement between human and animal diagnosis. In our conclusion we will discuss how our comparative analysis may underline possibilities both for the emerging sociology of veterinary medicine, and for the broader field of the sociology of diagnosis.

### Diagnosis and professional power

Balint famously wrote that diagnosis was a matter of organisation and negotiation: The patient presented a picture of disarray to the doctor; the diagnosis made sense of this disarray. The most pressing need for the patient, even before therapy, is ‘*the request for the name of their illness* (italics in the original)’ (Balint 1964: 25). His work underlined the profoundly social moment of diagnosis, positioning the roles of patient and clinician, and describing the negotiation which takes place. To date, there is little existing sociological research on diagnosis in veterinary practice, apart from studies on the underlying mechanisms of clinical reasoning (Cockcroft 1998), or how euthanasia is negotiated after diagnoses (Morris 2012a). Here, we contend that diagnosis also represents a powerful social moment in veterinary practice.

In human health, the ability to diagnose is the medical preserve and is thus a key marker of professional expertise. When other health professionals (nurse practitioners and physiotherapists) have diagnostic rights, this is within a limited scope of practice, and has often been achieved as a result of concerted lobbying and negotiation (Keeling 2015). In UK veterinary medicine, diagnosis is considered an act of veterinary surgery, so can only be carried out by a fully qualified veterinarian, rather than a veterinary nurse or paraprofessional. Indeed, the 1966 Veterinary Surgeon's Act positions diagnosis at the core of veterinary practice: The Act delineates the tasks of veterinary surgery as including (in order) ‘(i) *the diagnosis of diseases in, and injuries to, animals including tests performed on animals for diagnostic purposes*; (ii) the giving of advice based upon such diagnosis; (iii) the medical or surgical treatment of animals; and (iv) the performance of surgical operations on animals’ (Veterinary Surgeons Act 1966: emphasis added).

As in human medicine (Berger 2013), the act of diagnosis in veterinary medicine also helps confirm the identity of the veterinarian as scientist. As graphically described by Hamilton (following Latour) in relation to farm animal practice; ‘Moving from the nose to the tail, the vet's clinical examination pares down the mess of the cow's body, translating its smells, muck, and foul‐smelling fluids into ‘clean’ scientific discourse’ (Hamilton 2007: 491). Detailed ethnographic work such as Hamilton's is still relatively rare (Clarke and Knights 2018), but has shown the way in which diagnosis can be more of an art form, than a strict process of logic, where the mess of sensory information is transformed into diagnosis or ‘fact’.

Not only does diagnosis confirm the scientific and professional power of veterinarians *vis a vis* other para‐professionals, it is also important in maintaining an expertise boundary between veterinarians and animal owners. This is made clear in the UK Code of Conduct for Veterinary Surgeons which describes how veterinary surgeons and nurses should be attentive to, and note, client complaints in the medical record. ‘It should be noted, however’ the Code of Conduct warns ‘that diagnosis and clinical opinion is a matter of clinical judgement and should not be changed solely at the client's request’ (Royal College of Veterinary Surgeons 2012).

While such reference to the accuracy of records may at first sound like a technical matter, the above quote confirms the continued importance of diagnosis as the preserve and signifier of professional power and authority. However, despite this regulatory attempt at professional boundary‐work, there are also challenges to this power and prestige. As we now discuss, clients also have a key role in the diagnosis process.

### Diagnosis and lay contestation

In comparison to the power dynamics of doctor–patient relationships, veterinarian–client relations remain relatively unexamined (Morris 2012a: 178). This makes the veterinary profession ripe for detailed, sociological analysis. Indeed, the profession itself exemplifies the cultural ambiguity of animals as both subjects and objects (Morris 2012a: 6), with the veterinary encounter operating as a complex triad (Grimm *et al*. 2018) of patient, veterinarian and animal owner. Within this context, animal owners are normally entitled to decide whether to assent to therapeutic directions proposed by the veterinarian, unless the situation relates to notifiable diseases (May 2013: 50). Letting owners decide, however, may lead to suffering or even the death of the animal. This situation may weigh heavily on the shoulders of veterinarians. Most seek, and indeed make an oath to, prioritise animal welfare ‘above all else’ (Royal College of Veterinary Surgeons 2012). This means that to practice veterinary medicine with animal welfare at the fore, and owner decision‐making as a right, involves a highly complicated process of negotiation (see Morris 2012a), where veterinarians are ‘enmeshed in a web of moral duties and obligations that can and often do conflict’ (Rollin 2006: 15).

In terms of lay expertise, empirical studies are starting to show the way in which animal owners assert their views. In the example of companion animals, Burrow (2017: 358) argues that ‘clients, because of their close day‐to‐day proximately with their pets, have an intricate and detailed understanding of their pet's behaviour’. Based on a study using conversation analysis, he claims that this expertise can and is used to challenge the veterinarians’ claim to authority. Similarly, Degeling's historical work (2009: 399) identifies a gradual acceptance by orthopaedic vets of ‘owner's accounts of the relative welfare of the interior selves of their animal patients’.

Burrow, and others, including the British Veterinary Association (2016) have likened veterinary medicine to paediatrics (and see Fettman and Rollin 2002), in that parents make decisions on behalf of their children, and animal owners on behalf of their pets. However, we would caution against this direct comparison. As argued by scholars working on informed consent (Ashall *et al*. 2018, Gray *et al*. 2018), the underlying ethical principle in the two clinical domains is different:

Whilst medical consent protects a patient's rights to make autonomous decisions concerning their own body, veterinary informed consent aims to protect an owner's right to make autonomous decisions concerning their legal property (Ashall *et al*. 2018).

Another key source of contestation in relation to diagnosis relates to finances. As Morris has argued, ‘unlike in most doctor–patient interactions, frank discussions of cost of care as it relates to life‐and‐death issues are common in veterinary‐client relationships’ (2012a: 11). In the human field, health insurance or national health services are likely to foot the bill for care. Veterinary medicine, in contrast, operates as a contractual service, where client ability and willingness to pay contributes to diagnosis operating as a complex dance. This dance involves the veterinarian making a series of assumptions about both the client and the animal patient. For example, in terms of the former:

Bare bones’ clients might be given less than ideal options, with detailed financial estimates, that afford an animal minimal care. Conversely, ‘top notch’ clients may be given information only about premium care options with less emphasis on financial estimates (Morgan 2009: 78).

However, it is also not as simple as maximising diagnostic tests and treatments for those clients perceived as more willing to pay, or with insurance cover for their animal. As Morgan argues, vets in her qualitative study reported that they felt ‘uncomfortable encouraging clients to consent to therapies or diagnostic tests in older animals, even when the client might be willing and able to pay for the services’ (Morgan 2009: 52). This was echoed in Morris’ study of American vets, with one noting that *There is such a thing as a client having too much money* (Morris 2012a: 11). This might be in cases where the vet believes that the treatment or diagnostic tests are not in the best interests of the animal. In other words, in veterinary medicine there is sometimes a willingness to acknowledge the limits of diagnosis for achieving the best outcome. In human medicine, diagnostic uncertainty is less likely to be tolerated (Jones 2016), and all symptoms must be explained, even where the diagnosis may not result in a better outcome than management would have already provided, or may cause ethical problems of overdiagnosis (Carter *et al*. 2016).

The complex dance performed by vets and clients is also influenced by images of the value (or not) of the particular animal. So, regardless of welfare, some vets may believe that ‘clients who pursue intensive diagnostics and treatments are wasting their resources’ (Morgan 2009: 125), so base their actions on their own ethical attitude to animals. Another study confirms this, finding that ‘veterinarians may fail to offer extensive diagnostics on a hamster because they consider the animal to be less morally important (or assume that the client does) than a dog or cat’ (Morgan and McDonald 2007: 167).

Conversely, veterinarians may wish to offer diagnostic services but worry that they will be perceived as using diagnosis as a way to raise money for their practice. This is perhaps even more acute in veterinary than in human medicine, with some, in a US context, claiming that vets find it difficult to raise the topic of money fearing this may pollute their image: This anxiety makes sense, particularly if we appreciate that in order for occupational groups to be considered fully professionalised they need to distinguish their professional work from a purely business model (Morris 2009: 39).

We know that clients expect us to demonstrate affection for their animal, and we fear that financial discussions may somehow diminish the perception of our love for and dedication to animals (Klingborg and Klingborg 2007: 80).

Research using conversation analysis is starting to find evidence of ‘interactional sensitivity’ caused by this clash of business and medical models of veterinary services (MacMartin *et al*. 2018: 528). More specifically, work by Stivers in Southern California shows how companion animal owners participate in what she identifies as ‘pre‐diagnostic commentary’. During this phase before the ‘official diagnosis’, vets and clients are effectively ‘negotiating the range of diagnostic possibilities’, including taking into account the clients willingness to pay (Stivers 1998: 264). This finding appears to contrast with the Code of Conduct cited above, which asserts that diagnosis is the sole preserve of the clinician.

In summary, then, the triad of vet–owner–patient and the role of finances present a challenge to simplistic understandings of lay‐expert roles in veterinary practice and in diagnosis. In principle, diagnostic direction and therapeutic avenues may be the preserve of the professional, as in human medicine, but animal owners arguably retain more power in the diagnosis process. Morgan (2009: 65) summarised this neatly, writing: ‘Clients set the stage for the care of the patient by choosing when to seek the advice or services of a veterinarian, by authorising diagnostic or treatments plans (or not), and by continuing treatments and recommendations initiated by the veterinarian’.

Finally, it is possible that lay influence over the medical encounter is increasing over time. Instructions on being a patient in the nineteenth century required submission to the physician's authority: ‘The obedience of a patient to the prescriptions and instructions of his medical adviser should be prompt and implicit, and his attention to them uninfluenced by his own or other crude opinions, as to their fitness’ (Styrap 1878: 45). A shift away from this model of interaction, characterised by contemporary thinking as ‘paternalistic’ focuses instead on patient autonomy and expertise, as well as healthcare consumerism.

A similar trend away from paternalism has been discussed in the veterinary medicine (Gray *et al*. 2018, Kogan *et al*. 2009). One identified cause is the increasing use of the Internet which is ‘transforming human and animal health and the relationships that people have with their healthcare providers’ (Kogan *et al*. 2009: 3), the e‐scaping we referred to above. While the shift from paternalism to client‐focused care is often cited, on the ground, the picture is not actually clear‐cut in either human (Fox and Ward 2006, Lupton 1997) or animal medicine (Bard *et al*. 2017). Indeed, a recent study with veterinary professionals found that some see their role as providing their own opinion on the best course of action, whereas others feared taking this more paternalistic role (Armitage‐Chan *et al*. 2016).

### Diagnosis and interspecies relations

If we can study diagnosis as a moment for, and signifier of, professional power, and a route into understanding lay‐expert contestation, we here propose that it can also be a lens through which to understand and appreciate interspecies health relations.

For those working in the veterinary profession, sociological reference to ‘interspecies health relations’ would merely be regarded as an empirical description of their lived reality of occupational practice: In most spheres of work, veterinarians are constantly moving between animal species, and applying expertise from one to another to aid with diagnosis. Indeed, the ‘cascade’ system allows them to use clinical judgement to use drugs licensed for another animal species or, if not available, a drug licensed for human use (Gov.uk 2015). The literature also contains isolated reports of vets prescribing human drugs for animals (Rock and Babinec 2008: 333) and, historically, of vets treating human patients (Stivers 1998, Woods and Bresalier 2014). Vets are also physically involved in utilising a whole host of technologies originally designed for humans including ‘medical, dental, and surgical care, including dialysis, root canals, hip replacements, chemotherapy, cataract extractions, and even pacemakers’ (Morris 2012a: 181). Degeling's work also reminds us that this is not a recent phenomenon, and that technologies have also migrated from use in animals to humans. As he also notes, vets have historically resorted to using human analogies, given the impossibility of accessing the private experiences of their animal patients (Degeling 2009: 380).

For animal owners too, it is likely that they implicitly or explicitly draw on their own illness experiences when dealing with their animal's diagnoses or treatment. Empirical research on this interplay is still emergent, but isolated examples exist in the published social scientific literature, thus far in the companion animal sphere. For example, Rock and Babinec draw on interviews in relation to diabetes with pet owners in Canada and argue that owners simply accepted that ‘the same biomedical category could apply to cats, dogs and people’ (Rock and Babinec 2008: 335), and that ‘illness prototypes’ cross species boundaries. For example, Joe reported how he self‐diagnosed his dog *I watched him, and he was real sluggish, and he was going out to pee a lot, and drinking a lot of water. And, see, cause I got diabetes, I know that's what you do*. Likewise, Darleen, who also has diabetes expressed relief at her dog Cooper's diagnosis *it was just diabetes, and I thought, oh, I can manage that, I'm managing myself. So I was actually quite relieved* (Rock and Babinec 2010: 16). Indeed, before receiving this news from the vet, Darleen did her own kind of lay diagnosis by appropriating technology meant for human health care. *And I just tested his sugar on my glucose reader … Yup. I did my own check* (Rock and Babinec 2010: 15). As the authors neatly summarise, ‘previous experience in relation to human health problems may influence how people respond to health problems in their companion animals, and *vice‐versa*’ (p. 17).

Similarly, in his social constructionist study of identity, Burrow quotes a client as describing her cat as ‘constipated’ (Burrow 2017: 365). Burrow admits that this lay ‘naming’ of a specific condition was not a common feature of his dataset, perhaps, as we have discussed above in relation to diagnosis and power, due to its potential for ‘encroaching into the professional's sphere of expertise’ (Burrow 2017: 366). Nevertheless, this research does suggest the possibility of lay interspecies health diagnosis.

Indeed, this idea seems plausible, given wider published evidence of the way in which human health behaviour impacts on animals’ health and illness. Examples include both participation and rejection of health technologies. In relation to the former, Ashall and Hobson‐West (2017) found that owners who donate blood themselves see it as a responsibility that their pet also donates blood, due to the kin status (Charles 2014) of their animal. In contrast, in relation to rejection, recent poll data have led the British Veterinary Association to express concern about spillover from human vaccination critique into the veterinary vaccination clinic (British Veterinary Association 2019).

These examples highlight how experiences in human health care may impact animal health care. However, the contrary also holds, so that experiences with animal health and diagnosis could impact our experiences of human health care. For example, Rock and Babinec cite reports of people claiming improvement in their own management of diabetes after managing their dog's diabetes. This interplay extended to more spiritual accounts, with one owner noting that *people say sometimes, you know, your dog gets the same disease you have because it helps you cope with it* (Rock and Babinec 2010: 16). This interplay can even be visceral or embodied, in the example of the Swedish cat owner whose stomach ‘cramped’ in response to hearing her terminally ill animal cough (Redmalm 2015: 29). Such spillover can also be relevant to death as well as life: Those defending human euthanasia will often refer to animal euthanasia as a point of reference (Hurn and Badman‐King 2019: 140). Indeed, we experienced this during the writing of this article when a middle‐aged woman we knew who had recently kissed her old dog goodbye told us, *I wish when it came to my time, they'd let me just have a pat on the head, and a send‐off*.

In summary, there are important parallels between human and animal medicine, but divergences as well, which should interest sociologists of medicine and of health. Of course, not all humans have pets or interact regularly with animals, and not all owner–animal relations are comparable, so care must be exercised in drawing wider claims about interspecies relations. However, for some cases, where the diagnoses (e.g. diabetes), technologies (e.g. vaccines) or healthcare processes (e.g. blood donation) are similar in human and animal medicine, we believe there is significant value in researching health and health decisions beyond species boundaries. Indeed, this work may become more urgent given the e‐scaped nature of medicine (Nettleton 2004). With the wide availability of health information, it is likely that owners seeking information to aid with the lay diagnosis of their pet or even their farm animal may also source and evaluate information on human health, further destabilising the porous boundary between human and animal diagnosis.

## Conclusions

As we have discussed, diagnosis forms a crucial aspect of veterinary care: It encapsulates professional power, has significant consequences for lay–expert interaction and serves as a powerful example of interspecies health relations. In making diagnoses and diagnostic plans, veterinarians are making intricate professional and ethical judgements about animal welfare, their relationship with the client, the client's relationship with their animal, the ethical standing of certain species, the finances of their practice, their own emotions if definitive diagnoses are not possible and who gets to call the shots. They are also required to juggle these responsibilities against a backdrop of a veterinary professional identity dominated by images of perfectionism (Clarke and Knights 2018, Knights and Clarke 2018) or the ‘infallible expert’ (Armitage‐Chan *et al*. 2016).

As we have started to highlight, this already complicated role is troubled in contemporary practice by the Internet which is undoubtedly causing professional (Kogan *et al*. 2009, Schupe 2015, Vet Record 2011) and individual concern. For example, one American vet complained that: *The degree to which clients have been willing to trust anything they read on the internet has been a bit demoralizing. Frequently even long‐term relationship clients will second guess my recommendation based on internet information* … (Kogan *et al*. 2009: 13–4).

The parallels with human medicine here are striking, in terms of professional anxiety about the impact of technology, and in attempts to retain power by influencing how clients interact with online information. However, what is dramatically different is the current lack of empirical social scientific work to study this topic in detail, and explore whether and how this has shifted the power balance between professional and clients, and how diagnosis has changed over time. Of the work that is published, what strikes us overall is the relative dominance of research based on studies in the companion animal field. We believe there would be a benefit to animal health and veterinary practice if research were brought to explore diagnosis in other veterinary contexts, on the role of insurance and also on the more ‘mundane’ examples of diagnosis. While work on end of life care and euthanasia (e.g. Morris 2012a) has been ground‐breaking for sociology, even work on discussions about diet (MacMartin *et al*. 2018) has shown how interactively dense veterinary consults can be.

Our primary aim in this article was to apply insights from the sociology of diagnosis to veterinary medicine. To our knowledge, this represents a novel task in itself. The contemporary veterinary profession faces many challenges (Armitage‐Chan *et al*. 2016), including a changing relationship with the state (at least in the UK), and arguably, a social shift in the relations between humans and animals (Hobson‐West and Timmons 2016). Within this context, we would urge the veterinary profession, and those with whom they collaborate, to make use of the detailed social scientific research on diagnosis, before rushing to judgement about how best to respond to the perceived challenges (or opportunities) of the Internet and the evolving vet–patient–human triad. More broadly, for veterinarians inspired by the current energy around the One Health agenda, we would also encourage greater awareness of the historical interplay between human and veterinary medicine (Woods and Bresalier 2014).

Our analysis also has relevance for human medical practitioners. In 2010, Blue and Babinec argued that ‘researchers may find that asking people about pet health problems represents a useful way of gaining an insight into experiences, expectations, attitudes, knowledge, and values pertaining to human health’ (Rock and Babinec 2010: 18). Our own exploration of interspecies health relations confirms that this claim is also likely to hold true for diagnosis. First, our argument could therefore be harnessed by public health promoters, with the previous caveat that, not all patients regularly interact with animals, and not all diseases cross species boundaries. Second, delving into veterinary medicine may serve as a useful reminder to human medics to prioritise the patient rather than the diagnosis. As we have discussed, veterinary medicine does at least attempt a commitment to the animal's welfare, despite the multiple obstacles to achieving this in every case.

For sociologists of health and illness, we hope that this article will encourage more work on diagnosis, for example, on the role of commercial drivers. Diagnosis does not occur in a vacuum, and we have shown how the commercial aspects of the owner–vet relationship are crucial for how diagnosis operates. However, as we have also shown, it is not the case that, after a clear moment of diagnosis, what matters is simply whether clients can afford the treatment. On the contrary, we have shown that the issue is multifaceted. This analysis is particularly timely, given the increasing commercial factors at play in human health care. As Conrad argues ‘In a culture of increasingly market‐driven medicine, consumers, biotechnological corporations, and medical services interact in complex ways that affect social norms in changing definitions of behaviours and interventions’ (Conrad 2005: 11). In this context, animal medicine therefore gives us a particularly salient example of consumer behaviour which could help us prepare for a future human medicine which may even bypass formal diagnosis in favour of lay‐determined goals and limitations.

For example, future work could focus on the way in which disease awareness campaigns are sometimes launched by industry players who stand to benefit from sales of treatments. Individuals may also see self‐diagnosis tests on web pages and in magazines (Ebeling 2011), or use apps which were designed by the gaming industry, rather than by any diagnostic experts (Lupton and Jutel 2015). Overall, more research is required to understand these commercial drivers, and their impact on ideals of welfare, communication and care.

Beyond diagnosis *per se*, we also hope that our analysis encourages more sociological work on animals and on veterinary practice. To return to where we started, Brown and Nading (2019: 6) argue that for medical anthropology ‘a view of health as more than human productively disturbs existing disciplinary settlements’. We hope this is also true for medical sociology, and that we can start to move beyond the speciesism that still dominates our field (Ashall and Hobson‐West 2017, Hobson‐West and Timmons 2016). Of course, the best methods and approaches to use are up for debate: Some scholars will wish to keep the human as their unit of analysis but, as we have done, explore how our interactions with animals and animal health care cannot and should not be artificially excluded from how we research health and illness practices. For others, it is more important to focus on the co‐evolution of species (Haraway 2003), or even the multispecies relations inside human bodies (see Brown and Nading 2019). Whatever approach is taken, the result will be a greater appreciation of the interdependencies of human and animal medicine. As summarised by Brown and Nading, ‘The road to knowing and visualizing the diseased human body, then, has been historically routed through the animal body, and *vice‐versa*’ (Brown and Nading 2019: 12). This persists today (Davies *et al*. 2016), as contemporary laboratory science continues to rely on animal bodies to produce new knowledge, new medicine and new diagnoses for both human and animal patients.
